# Trend, spatial distribution, and factors associated with HIV testing uptake among pregnant women in Ethiopia, based on 2005–2016 Ethiopia demographic and health survey: A multivariate decomposition analysis and geographically weighted regression

**DOI:** 10.1371/journal.pone.0308167

**Published:** 2024-10-04

**Authors:** Betelhem Abebe Andargie, Emebet Birhanu Lealem, Dessie Abebaw Angaw

**Affiliations:** Department of Epidemiology and Biostatistics, Institute of Public Health, College of Medicine and Health Sciences, University of Gondar, Gondar, Ethiopia; Ethiopian Public Health Institute, ETHIOPIA

## Abstract

**Introduction:**

HIV testing during pregnancy is an integral component and first step of prevention for mother to child transmission, initiation of antiretroviral treatment and diagnosis of HIV/AIDS. However, Ethiopia and other sub-Saharan African countries face challenges in meeting the first target of the 95-95-95 global initiatives. This study examines trends, spatial distribution, and factors influencing HIV testing among pregnant women in Ethiopia from 2005 to 2016, using data from the Ethiopia Demographic and Health Surveys.

**Methods:**

The study was based on three consecutive demographic and health survey in Ethiopia. A total weighted sample of 13,020 women who gave birth within 2 year proceeding each survey year was included in each survey. Logit based decomposition analysis technique was employed to identify factors contributing to the change in HIV testing uptake among pregnant women overtime. ArcGIS version 10.7.1 and SaT Scan version 10.1software were used for the spatial analysis and geographically weighted regression.

**Results:**

HIV testing uptake among pregnant women has significantly increased from 0.51% in 2005 to 32.4% in 2016 with 2.9% annual rate of increment in Ethiopia. About 75.9% of the overall increase in HIV testing uptake among pregnant from 2005–2016 was due to increases in women’s composition with knowledge of Mother to child transmission of HIV (3.2%), HIV counseling (10.3%), 4 or more antenatal care visits (31.4%), health facility delivery (6.3%), not perceiving distance from the health facility as a big problem (1.1%), and urban residence (0.6%). Spatial variation of low proportion of HIV testing was non-random in all three surveys (Moran index, p-value<0.05). Hot spot clusters exhibited in all the three waves includes Tigray and SNNPRs in 2005 and consistent hotspot areas in Benishangul-Gumuz, Somali, SNNPR, and Gambella in 2011 and 2016 EDHS. Lack of knowledge of Mother to child transmission of HIV, lack of antenatal care visit, lack of media exposure, and health facility delivery were significant predictors for the spatial variation of low proportion of HIV testing uptake across regions in Ethiopia in 2016.

**Conclusion and recommendation:**

Over all, there has been a substantial increase in HIV testing uptake among pregnant women overtime in Ethiopia, but it still far away from achieving the 2025 HIV testing targets. Knowledge of Mother to child transmission of HIV, HIV counseling, Number of Antenatal care visit, previous place of delivery, residence and distance to health facility were significant contributing factors for the change in HIV testing uptake. There was geographical disparity in HIV testing uptake across regions in all three EDHS. Lack of knowledge of Mother to child transmission of HIV, lack of ANC visit, lack media exposure, and health facility delivery were significant predictors. Geographic-based interventions, together with broader public health strategies, are essential for advancing HIV testing uptake.

## Introduction

*Human immunodeficiency virus (HIV)* infection during pregnancy poses significant risks to maternal and child health, contributing to maternal morbidity, mortality, and pediatric HIV infections globally [1]. In 2021, approximately 1.3 million pregnant women worldwide were living with HIV, with sub-Saharan Africa bearing 90% of this burden and one quarter of pregnancy related deaths [2]. Ethiopia, in particular, faces significant challenges despite efforts to mitigate mother-to-child transmission (MTCT) of HIV, in 2018, HIV prevalence among pregnant women was reported at 5.74% [3]. Although MTCT declined from 39.55% in 2000 to 16.9% in 2019 in Ethiopia, still remains as one of the top ten countries in the world with the highest burden of HIV infections due to MTCT [4].

According to the World Health Organization (WHO) and the Centers for Disease Control and Prevention (CDC) HIV testing during pregnancy is recognized as an essential component and the initial step in preventing mother-to-child transmission PMTCT [5, 6]. It is recommended that every pregnant woman should be tested at least once during pregnancy to reduce any complication related to HIV [5]. The possibility of MTCT can be about 15–30% before and during pregnancy, but with pregnant women being tested for HIV and adhering to PMTCT protocols including timely initiation of antiretroviral therapy, safe delivery practices, and appropriate infant feeding strategies, the risk of MTCT will be 5% or lower [7]. Even if HIV testing is a proven strategy to PMTCT, countries in sub-Saharan Africa have been struggling to achieve the first target of Joint United Nations Programme on HIV/AIDS(UNAIDS) with HIV testing uptake only less than 90% [8].

According to Demographic health survey (DHS) data HIV testing uptake among pregnant women in sub-Saharan Africa countries, varies from 6.1% to 81.5% [9, 10]. In Ethiopia, this uptake varied in different part of the country through the years from 6.9% to 82.5% [11–14]. Factors influencing uptake include socioeconomic status, education, awareness of MTCT risks, sexual behavior, and media exposure [10, 15–20].

UNAIDS,UNICEF and Sustainable Development Goal (SDG) 3.3 as response to the global epidemics, has designed intervention to eliminate new infections of HIV through expanded PMTCT and testing pregnant women, end HIV/AIDS by 2030 [21, 22]. Ethiopian Federal HIV/AIDS Prevention and Control Office (FHAPCO) and Federal Ministry of Health guideline, pregnant women are one of the highly concern given population groups for HIV. And committed to increase number of pregnant women uptake of HIV to 95% and reduce MTCT to <5% by 2025 [4]. But, despite global and national efforts, in Ethiopia percentage of pregnant women receiving HIV testing hasn’t reached the global target so far.

Even though pregnant women’s HIV testing uptake has shown variation through time and regions in Ethiopia, previous evidence failed to assess the spatial distributions and the contribution of each of the variables for the observed changes over time [23–25]. Understanding these factors associated with the time and spatial variation will be important to prioritize HIV related programs that are targeted for pregnant women in the identified hotspot areas. Hence, this study aimed to fill the gap by investigating the trend, its source of change and spatial distribution and factors associated with geographical distribution of HIV testing uptake among pregnant women in Ethiopia using the Ethiopian demographic health survey.

## Methods and materials

### Data source

This study was based on secondary data that were collected for the 2005, 2011 and 2016 Ethiopian Demographic Health Survey (EDHS) which was obtained from USAID–DHS program data sets. Variables anticipated to be associated with HIV testing uptake among pregnant women were extracted from the ‘women dataset’, based on the reviewed literature, and then processed for further analyses.

### Study design and period

The study was done using repeated cross sectional study in three consecutive EDHS time periods between 2005–2016. EDHS is population based nationally representative study. The data collection period of each survey was April 27 to August 30 in 2005, December 27 in 2010 to June 3 in 2011 and from January 18 to June 27 in 2016 [23–25].

### Study area

Ethiopia is located in the Horn of Africa at 3′ and 14.8″ latitude 33′ and 48′ longitude bordering Somalia, Sudan, Djibouti, Kenya and Eritrea. At the time of the survey, Ethiopia was sub-divided into nine regional states and two administrative cities. The regional states are Tigray, Afar, Amhara, Oromia, Somali, Benshangul-Gumuz, Southern Nations, Nationalities, and Peoples’ Region (SNNPR), Gambela, and Harari. The administrative cities were Addis Ababa and Dire-Dewa. Each region is divided in to zones and zones into administrative units called woredas and each woreda is classified into simplest administrative unit called kebeles. It has 68 zones, 817 districts and 16,253 kebeles Regarding the health system of Ethiopia, based on the health sector transformation plan the health care system of Ethiopia is organized three tier health system: the first at district level is primary health care unit (PHCU) consists of health posts, health center and primary hospitals while the second level include secondary care consists of general hospitals and specialty centers: tertiary care consists of comprehensive specialized hospital. The health extension program also plays key role in increasing service utilizations [26].

### Source and study population

The source population for this study was all reproductive-aged(15–49) women who gave birth within 2 years prior to the 2005,2011,2016 EDHS in Ethiopia, Whereas the study population was all reproductive aged women aged (15–49) who gave birth within 2 years prior each survey in the selected enumeration areas in Ethiopia. Women who gave birth within 2 years prior to each survey within enumeration areas with zero geographic coordinates and no proportion record were excluded for spatial analysis.

### Sample size determination and sampling procedure

The Ethiopia demographic health surveys have considered different parameters to estimate the final sample size. The detailed sample size determination can be found in each report [23–25]. For this study women (IR file) was used to extract reproductive aged women who gave birth 2 years preceding the survey. Thus, this study included total weighted sample size of 4321 in 2005, 4453 in 2011 and 4246 in 2016.

A Stratified-clustered two-stages sampling was used to select the respondents. The 1994 Population and Housing Census provided the sampling frame from which the 2005 sample were drawn, and the 2007 Population and Housing Census provided frame for both the 2011and 2016 EDHS. Each district was stratified into urban and rural areas. Then Samples of EAs were selected independently in each stratum in two stages. The first stage used in each survey being the enumeration areas consisting of urban and rural areas. The second stage used in the survey is households with an equal probability systematic selection. Each survey had a cluster size of 540 in 2005, 624 in 2011 and 645 in 2016 EDHS. Accordingly 24–32 households were selected from each clusters and the interview was completed for with 14,070 women in 2005, 16,515 in 2011, 15,683 in 2016 [23–25].

### Study variables

The outcome variable was HIV testing uptake among pregnant women, coded as a binary variable (“tested for HIV” = 1 and “not tested for HIV” = 0); whereas Socio-demographic variables (Age, Educational status, paternal educational status, Wealth status, marital status, Occupational status. Media exposure. Residence, Region), HIV/AIDS and sexual behavior related variables (Knowledge of MTCT of HIV, HIV counseling, Early sexual initiation, Risky sexual behavior, Comprehensive knowledge of HIV AIDS) and Health related variables(Number of ANC, Parity, pregnancy wanted, place of delivery, healthcare decision making autonomy, Distance to Health facility) were considered as independent variables.

### Operational definitions

**HIV testing uptake**: Pregnant women were characterized as having HIV testing uptake if women tested for HIV during her recent pregnancy and received the result according to DHS guideline [27].

**HIV Counseling:** If women received counseling on HIV during recent pregnancy, in relation to HIV transmission from mother to child, how to prevent HIV transmission, and getting tested. If the respondent was counseled on three topics, it was categorized as “yes”, otherwise “No” [27].

**Knowledge of MTCT HIV:** Defined as awareness of women about the possibility of HIV transmission from HIV positive mother to her child, which is generated from three questions knowledge about HIV transmission during breast feeding, during delivery, and pregnancy. If the respondent responded on three topics, it will be categorized as “yes”, otherwise “No” [27].

**Comprehensive knowledge about HIV**: assessed based on three questions related to HIV prevention and three questions related to the modes of HIV transmission and graded as low (if a woman answered ≤3 questions correctly), high (if a woman answered ≥ 4questions correctly) [27].

**Wealth status:** Wealth index was constructed using household selected asset data through principal component analysis to categorize individual in poorest, poorer, middle, richer and richest [27].

**Media exposure**: assessed by combining whether women had any of these media options (Radio, TV, Newspaper and magazine),categorized as yes (if a woman had been exposed to at least one of these media) and no (if women were not exposed to at least one of the media) [27].

**Early sexual initiation:** defined as the experience of first intercourse before 18 years of age [28].

**Risky sexual behavior:** assessed based on the four questions. It was categorized as “no risk” if (score 0), “with risk” if (score ≥ 1) [29].

**Health care decision making autonomy:** Women who take health care decision alone or with their partner represented a woman with health care decision making autonomy, while women where other person alone decides for health care, represents a woman with no health care decision making autonomy [30].

### Data collection procedure

Data in EDHS for HIV testing uptake was collected since 2005 and 2 consecutive surveys after that in nine regions and two administrative cities. The questionnaire for each EDHS was adopted from the Measure DHS project to reflect the health issues in Ethiopia. This study was based on the EDHS data collected using the woman’s standard questionnaire. The DHS questionnaire was first prepared in English then translated to three major languages Amharic, Affan Oromo, and Tigrigna. Along all the EDHS’s data collectors were recruited based on language skills, academic qualifications and previous survey experience and trained for collection. The data were collected through interviewing technique and fieldwork procedures [23–25].

### Data process and analysis

STATA 17 software was used for data extraction, cleaning and appropriate statistical analysis on individual women records (IR). Before further analysis missing values of the outcome was counted as not having received testing during pregnancy based on the DHS guideline. Data was weighted before any statistical analysis using women’s sampling weight, primary sampling unit, and strata using the “*svy*set” Stata command to restore representativeness of the data and to get reliable statistical estimates. Then, Appending of the cleaned 2005, 2011 and 2016 data sets were done for the Decomposition analysis.

#### Trend and decomposition analysis

The trends for HIV testing uptake were examined by descriptive analysis separately dividing EDHS into three phases for the periods 2005–2016: first phase (2005–2011), second phase (2011–2016) and third phase (2005–2016) to show change in proportion of HIV testing uptake over time. A p-value less than 0.05 and the 95% Confidence Interval (CI) were used to declare a significant change of the trend.

*Logit* based decomposition analysis was employed to identify the contributing factors to change in the proportion of HIV testing uptake between EDHS 2005–2016. It used the output of logistic regression analysis to divide the observed difference in HIV testing uptake between the surveys in to components. Decomposition analysis was done using the “mvdcmp” command of STATA version 17 [31]. The decomposition model additively explains this difference in HIV testing uptake over time by **Endowments** (i.e. the change in HIV testing uptake is due to the difference in the composition changes or characteristics of surveys, which is explained) and **Coefficients (i.e.** the change in HIV testing uptake due to the effects of those selected explanatory variable (characteristics) that are unexplained).

Let the 2016 EDHS and reference 2005 EDHS datasets be denoted by A and B respectively, For logistic regression, the log-odds or logit of HIV testing uptake is taken as:

Logit(A)−Logit(B)=F(XAβA)−F(XBβB)

$$=\underset{E}{\underset{︸}{F(XA\beta A)-(XB\beta A)}}+\underset{C}{\underset{︸}{F(XB\beta A)-F(XB\beta B)}}$$

#### E C

The “E” represents endowments, which is usually the explained component or by characteristics; Whereas “C” component denote coefficients or effect of characteristics which is unexplained representing the change in HIV testing uptake due to the effect of predictor variables.

Finally, variables with a *p*-value less than 0.2 from the bi-variable decomposition analysis were selected as candidate variable for multivariable decomposition analysis. finally, in the multivariable analysis p-value <0.05 and the corresponding coefficient β with 95% confidence interval(CI) was used to state the significant factor for the change in HIV testing uptake among pregnant women.

#### Spatial distribution

Multiple Spatial analysis was used to analyze the geographical variation of low proportion of HIV testing uptake among pregnant women in EDHS clusters of in 2005, 2011 and 2016. All the analysis was done at regional level and the unit of this spatial analysis were the cluster. ArcGIS software version 10.7.1 was used for visualization, exploration and creation of Maps. The CSA (Central Statistical Agency) database provided the Ethiopian district delineation shape file.

#### Spatial autocorrelation

Spatial autocorrelation analysis was used for evaluating autocorrelation at regional level for each survey from 2005–2016 by taking the total data set and producing a single output value whose ranges were between− 1 to+ 1. value closes to − 1 indicates dispersed low proportion of HIV testing uptake, whereas value close to + 1 indicates clustered low proportion of HIV testing uptake and Moran’s I value of 0 indicates randomly distributed low proportion of HIV testing uptake among pregnant woman [32].

#### Hotspot analysis (Getis-OrdGi* statistic)

Hotspot statistics was computed to measure how spatial autocorrelation/ degree of clustering varied over the study location by calculating Getis-OrdGi* statistics for each area in each survey. Getis–Ord Gi* was calculated as a ratio of sum of the values in neighboring locations to the sum of all the values in the study area. Statistical output with high GI* indicated “hotspot” of low proportion HIV testing uptake whereas low GI* means a “cold spot of low proportion HIV testing uptake among pregnant women [33].

#### Spatial interpolation

It is very challenging to gather reliable data in each and every area of the country to know the burden of low proportion HIV testing uptake among pregnant women. Therefore, the spatial interpolation technique was used to predict HIV testing uptake in the unsampled areas in the country based on sampled enumeration areas in each survey. There were different deterministic and geostatistical interpolation methods. Among those methods, ordinary Kriging was used since it incorporates the spatial autocorrelation; it statistically optimizes the weight and had smallest root mean square error value and residuals for predictions of low proportion of HIV testing uptake among pregnant women in unobserved areas of Ethiopia.

#### Spatial scan (SaT Scan) statistical analysis

Kuldorff’s sat scan version 10.1 software was used. Pregnant women with low proportion HIV testing uptake was taken as cases and without it as controls to fit the Bernoulli model. The spatial scan statistics used a circular scanning window that moved across the study area. The scanning window with the maximum likelihood was the most likely performing cluster, and a p-value was assigned to each cluster using Monte Carlo hypothesis testing by comparing the rank of the maximum likelihood from the real data with the maximum likelihood from the random data sets. Under the null hypothesis, a large number of data sets were generated by randomly permuting the locations of observations. It was performed to determine whether or not the identified clusters were significant [34].

#### Ordinary least square analysis

The ordinary least square (OLS) analysis was done for the 2016 EDHS using variables that were found to be significant at different literatures and tend to have a differences geographically. The OLS model is a global model that predicts only one coefficient per independent variable over the entire research area. It assumes stationary or consistent relationship across the study area. Then, the model performance was checked. The OLS was used as a diagnostic tool and for selecting the appropriate predictors (concerning their relationship with HIV testing uptake) for the Geographic Weighted Regression (GWR) model. Findings from ordinary least squares (OLS) regression were only trustworthy if the regression model satisfies all of the assumptions that are required by this method. The Jarque-Bera test was used to assess the normality assumption for residuals. As residuals were not spatially auto-correlated, the statistically significant Koenker (BP) statistic showed that the relationships modeled are not consistent (either due to non-stationarity or heteroscedasticity). Multicolinearity (Variance Inflation Factor) was used to check relationship among predictor variables and VIF values less than 7.5 were declared as having no Multicolinearity, lastly coefficients have the expected sign and statically significant and strong adjusted R2 values [35]. The OLS regression equation [36]:

$$Yi=\beta +\sum_{k=1}^{p}\left(\beta kx_{ik}\right)+\epsilon i$$

Where i  =  1, 2…n; β0, β1, β2…βp are the model parameters, yi is the outcome variable for observation i, *X*_*ik*_ are explanatory variables and ∈_1_, ∈_2_…∈_*n*_ are the error term/residuals with zero mean and homogenous variance σ2.

#### Geographically weighted regression (GWR)

GWR is local spatial statistical technique that assumes the non-stationary relationship between the dependent and explanatory variables across EAs. The GWR analysis was done because the Koenker statistics was significant (p-value<0.01), which means the relationships between the dependent and the independent variable change from location to location. In the GWR analysis, the coefficients of the explanatory variables take different values across the study area. The GWR is computed as [37]:

$$Yi=\beta o(ui+vi)+\sum_{k=1}^{p}(uivi)x_{ik}+\epsilon i$$

Where, yi are observations of response y, *u*_*i*_*v*_*i*_ are geographical points (longitude, latitude), βk (uivi)(k  =  0, 1 … p) are p unknown functions of geographic locations *u*_*i*_*v*_*i*_, *X*_*ik*_ are explanatory variables at location *u*_*i*_*v*_*i*_, i  =  1, 2, … n and ∈i are error terms/residuals with zero mean and homogenous variance (σ2).

In this study, the corrected Akaike Information Criteria (AICc) and adjusted R-squared squared were used for model comparison of OLS and GWR model. A model with the lowest AICc value and a higher adjusted R-squared value was considered as the best-fitted model for the data. Finally, the coefficients which were created using GWR were mapped.

### Ethical consideration

Ethical clearance was obtained from university of Gondar, college of medicine and health science institutional review board. The approval letter for the use of the EDHS data was accessed after submitting the summary of the proposal from the Measure DHS https://dhsprogram.com/ Online for the 2005, 2011 and 2016 EDHS data with their geographical coordinates. Moreover, All Ethiopian Demographic and Health Surveys were conducted after obtaining ethical clearance from Ethiopia Health and Nutrition Research Institute Review Board, the Ministry of Science and Technology, Institutional Review Board of ICF International, and the CDC, and we confirm that the study was conducted according to the Declaration of Helsinki. GPS coordinate displacement was also performed on the actual locations of each cluster to maintain confidentiality of the surveyed respondent [23–25].

## Results

### Characteristics of study population

A total weighted sample of 13,020(4321 in EDHS 2005,4453 in 2011 and 4246 in 2016) women who gave birth in the past two year prior to each survey were included in this study. The result of this study revealed that about half of the respondents in all three EDHS were found within the age category of 20–29 years. Concerning women’s educational status, in survey 2005 more than three quarters (77.8%) of women’s were not educated, while it decreased to 66.4% and 60.4% in EDHS 2011 and 2016 respectively. Across the three consecutive surveys, the proportion of women with knowledge of MTCT of HIV increased from 24.0% in 2005 to 51.5% to 53.8% in 2016. Regarding ANC visit, more than two third women (71.4%) in 2005 had no antenatal visit, but decreased over time to 56.6% in 2011 to 35.3% in 2016 (**Table 1**).

**Table 1:** Characteristics of respondents in the 2005, 2011, and 2016 Ethiopian Demographic and Health Surveys.

Characteristics	Category	Weighted frequency (%) 2005 N = 4321	Weighted frequency(%) 2011 N = 4453	Weighted frequency(%) 2016 N = 4246
**Age**	15–19	365(8.4)	333(7.5)	279(6.6)
	20–24	1026(23.7)	1034(23.2)	972(22.9)
	25–29	1195(27.7)	1407(31.6)	1246(29.4)
	30–34	825(19.1)	788(17.7)	904(21.3)
	≥35	909(21.1)	891(20.0)	845(19.9)
**Religion**	Orthodox	1869(43.2)	1814(40.7)	1451(34.2)
	Muslim	1454(33.7)	1049(23.6)	1776(41.8)
	Other ^a^	998(23.1)	1590(35.7)	1019(24.0)
**Marital status**	Single	20(0.5)	38(0.9)	31(0.7)
	Married	4088(94.6)	4166(93.6)	4043(95.2)
	Others ^b^	213(4.9)	249(5.6)	172(4.1)
**Women’s education status**	No education	3363(77.8)	2956(66.4)	2566(60.4)
	Primary	768(17.8)	1296(29.1)	1302(30.7)
	Secondary and above	190(4.4)	201(4.5)	377(8.9)
**Paternal education status**	No education	2463(57.0)	2186(49.1)	2047(48.2)
	Primary	1379(31.9)	1866(41.9)	1617(38.1)
	Secondary and above	479(11.1)	401(9.0)	592(13.7)
**Occupational status**	Not working	3126(72.3)	2237(50.3)	2486(58.6)
	Working	1195(27.7)	2216(49.8)	1760(41.4)
**Wealth index**	Poorest	918(21.3)	1047(23.5)	994(23.4)
	poorer	926(21.4)	988(22.2)	925(21.8)
	Middle	957(22.2)	917(20.6)	881(20.8)
	Richer	859(19.9)	784(17.6)	788(18.6)
	Richest	660(15.3)	717(16.1)	658(15.5)
**Media exposure**	Yes	1621(37.5)	2631(59.1)	1780(41.9)
	No	2700(62.5)	1822(40.9)	24-6(58.1)
**Residence**	Rural	3999(92.5)	3846(86.4)	3733(87.9)
	Urban	322(7.5)	607(13.6)	513(12.1)
**Region**	Tigray	256(5.9)	273(6.1)	308(7.3)
	Afar	41(1.0)	40(0.9)	42(1.0)
	Amhara	1046(24.2)	983(22.1)	779(18.2)
	Oromia	1668(38.6)	1917(43.1)	1894(44.6)
	Somalia	168(3.9)	128(2.9)	178(4.2)
	Benishangul-Gumuz	40(0.9)	51(1.1)	44(1.0)
	SNNPRs	1005(23.3)	926(20.8)	855(20.1)
	Gambela	11(0.3)	15(0.3)	10(0.2)
	Harari	10(0.2)	11(0.2)	10(0.2)
	Addis Ababa	61(1.4)	95(2.1)	108(2.6)
	Dire-Dawa	15(0.3)	14(0.3)	18(0.4)
**Knowledge of MTCT of HIV**	Yes	1039(24.0)	2304(51.7)	2286(53.8)
	No	3282(76.0)	2149(48.3)	1960(46.2)
**Comprehensive knowledge of HIV**	Low	3686(85.3)	3269(81.5)	3057(72.0)
	High	635(16.7)	824(18.5)	1189(28.0)
**Early-sexual initiation**	<18 years	1594(36.9)	561(12.6)	2726(64.2)
	≥18 years	2726(63.1)	3892(87.4)	1520(35.8)
**Risky-sexual behavior**	No risk	502(11.6)	930(20.9)	812(19.1)
	With risk	3819(88.4)	3523(79.1)	3434(80.9)
**HIV Counseling**	Yes	65(1.5)	606(13.6)	994(23.4)
	No	4256(98.5)	3847(86.4)	3252(76.6)
**Knowledge of MTCT of HIV**	Yes	1039(24.0)	2304(51.7)	2286(53.8)
	No	3282(76.0)	2149(48.3)	1960(46.2)
**Number of ANC**	No visit	3086(71.4)	2521(56.6)	1499(35.3)
	1–3 visit	715(16.6)	1160(26.1)	1334(31.4)
	≥4 visit	520(12.0)	772(17.3)	1412(33.3)
**Parity**	1	777(18.0)	800(18.0)	869(20.5)
	2–4	1858(43.0)	1996(44.8)	1804(42.5)
	≥5	1686(39.0)	1657(37.2)	1573(37.0)
**Place of delivery**	Home	4024(93.1)	3916(87.9)	2631(62.0)
	Health facility	297(6.9)	537(12.1)	1615(38.0)
**Pregnancy wanted**	Wanted	3542(82.0)	3989(89.6)	3888(91.6)
	Not wanted	779(18.0)	464(10.4)	358(8.4)
**Distance to Health facility**	Big problem	3219(74.5)	3313(74.4)	2571(60.6)
	Not a big problem	1102(25.5)	1140(25.6)	1675(39.4)

Other ^a^*: protestant/catholic/traditional other ^b^*: Widowed, separated, divorced *MTCT: Mother to child transmission *ANC: Antenatal care

### The trends in HIV testing uptake among pregnant women over time

The overall proportion of HIV testing uptake over the study period has increased from 0.51% [0.32%, 0.77%] in 2005 to 19.98 [19.62, 20.36] in 2011 to 32.4% [31.0%, 33.8%] in 2016 with 2.9% annual rate of increment. The largest increase was seen in the first phase (2005–2011) with a 19.4% point change in HIV testing uptake, The change was substantially significant in all three phases with p-value <0.05 and non-overlapping 95% confidence intervals in each phase.

#### The trends in HIV testing uptake by women’s characteristics

**Fig 1:**
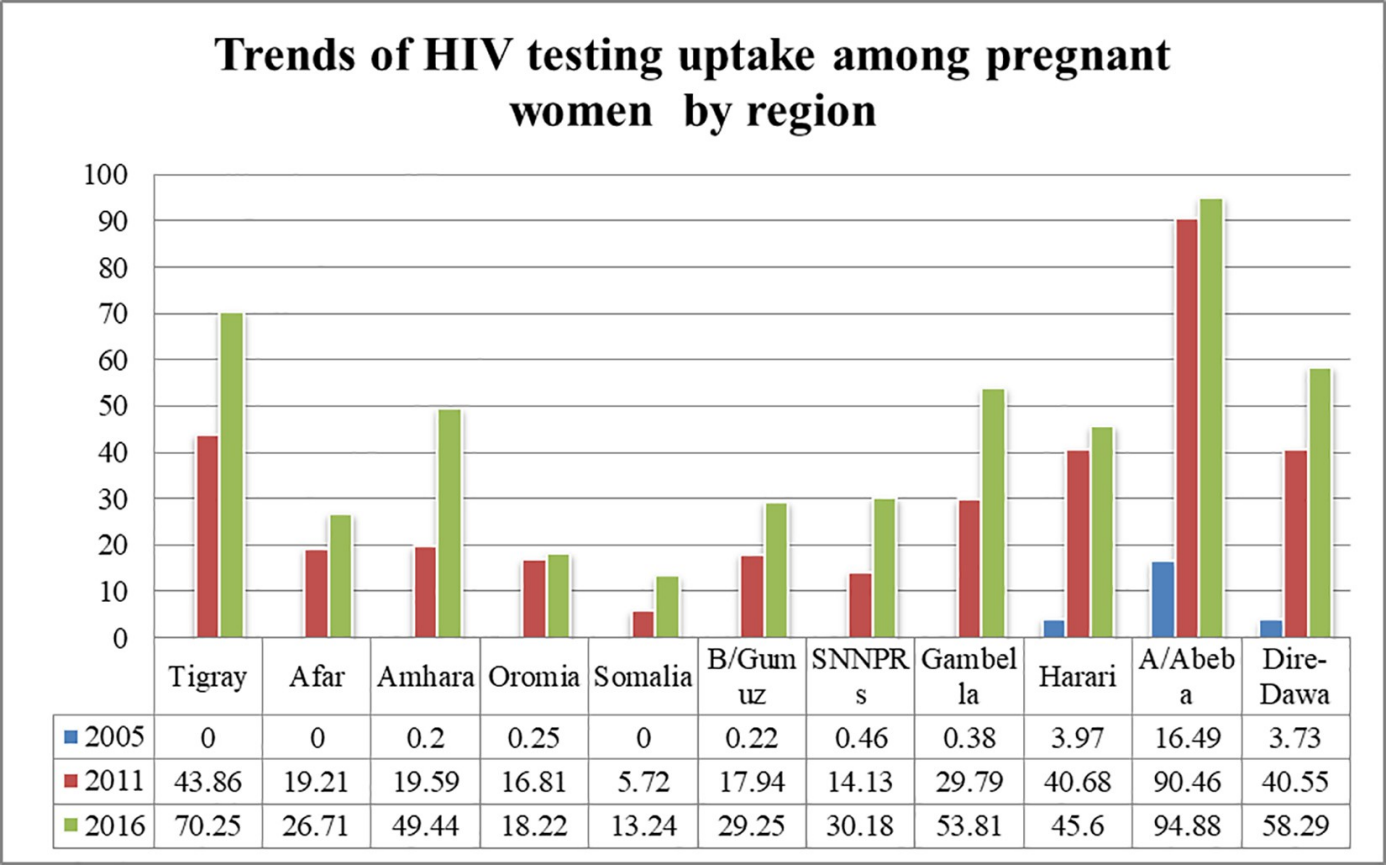
The trends in proportion of HIV testing uptake among pregnant women over time across regions of Ethiopia in 2005, 2011, 2016.

The trend in HIV testing uptake proportion among women’s who gave birth within two years prior to the survey showed variation according to their characteristics (S1 Table). The changes were positive in most of the categories. With regard to region the largest increment in the trend of HIV was shown in Adiss Abeba with a 70.3-point change in the third phase whereas the smallest increment was seen in Somali with only a 13.2 point change (**Fig 1**).

Regarding with the education status of women, the highest increment in HIV testing was in the first phase with 78.4 from 2005–2011 point change in secondary and above education. Concerning to number of ANC visits, trend in HIV testing uptake shown an increment with whose women who have four and above ANC visits, which increased from 2.56 in 2005 to 61.80 in 2016 (**S1 Table**).

### Decomposition analysis

#### Decomposition analysis of HIV testing uptake in Ethiopia, 2005–2016

Overall from 2005 to 2016, there was substantially significant increment in HIV testing uptake among pregnant women in Ethiopia. The decomposition result showed that both the change due to women’s composition (endowment) and change due to the effect of explanatory variable for the change in HIV testing uptake were significant (**Table 2**).

**Table 2:** Overall decomposition of change in HIV testing uptake among pregnant women in Ethiopia, 2005 to 2016.

HIV testing uptake	Coefficient	P-value	95% confidence interval (CI)	Percentage (pct.)
**E**	0.24219	<0.001	[0.23065,0.25372]	75.9**
**C**	0.076735	<0.001	[0.06617,0.0873]	24.1**
**R**	0.31892	<0.001	[0.30429,033356]	

#### Difference due to characteristics (endowment)

The decomposition analysis showed that about third-fourth (75.9%) of the increase in HIV testing uptake among pregnant women was attributed to the change in endowment between the survey periods keeping other factors constant. Number of ANC visit (31.4%), knowledge of MTCT of HIV (3.2%), HIV counseling (10.3%), previous place of delivery (6.3%), residence (0.6%) and distance to health facility (1.1%) showed statistically significant contribution for the change in the HIV testing uptake among pregnant woman (**Table 3**).

**Table 3:** Detailed decomposition analysis of change in HIV testing uptake among pregnant women in Ethiopia, 2005–2016.

Variables	Difference due to characteristics		Difference due to coefficient	
	Coeff (95% CI)	Pct.	Coeff (95% CI)	Pct.
Wealth status				
**Poorest**	1		1	
**Poorer**	0.0006145[-0.002365,0.001465]	0.2	0.003817[-0.005124,0.01275]	1.2
**Middle**	-0.00005331[-0.000015,0.0000027]	0.1	0.007146[-0.008113,0.022407]	2.24
**Richer**	0.0001137[-0.0000249,0.000252]	0.03	-0.001418[-0.008811,0.005974]	-0.44
**Richest**	-0.001237[-0.008483,-0.01095]	-0.21	0.006197[-0.001401,0,03797]	1.94
Educational status				
**No education**	1	0.7	1	-0.45
**Primary**	0.002241[-0.1013,0.005497]		-0.001445[-0.009551,0.06660]	
**Secondary and above**	0.001539[-0.0005360,0.003614]	0.48	-0.0001742[-0.002151,0,001803]	-0.05
Occupational status				
**Not working**	1	0.74	1	0.38
**Working**	0.002382[-0.00006121,0.005376]		0.001237[-0.008483,0.01095]	
Media exposure				
**Yes**	-0.0002216[-0.0009329,0.0004896]	-0.06	-0.002451[-0.01811,0.01320]	-0.76
**No**	1		1	
Knowledge of MTCT of HIV				
**Yes**	0.10524**[0.003912,0.17128]	3.3	-0.004670[-0.1365,0.004315]	-1.46
**No**	1		1	
HIV counseling				
**Yes**	0.03273**[0.2556,0.03990]	10.3	-0.0004004[-0.00096,0.000165]	-0.12
**No**	1		1	
Comprehensive HIV knowledge				
**Low**	1	-0.72	1	-0.61
**High**	-0.002305[-0.005647,0.001036]		-0.001972[-0.008577,0.004632]	
Risky sexual behavior				
**With risk**	0.002571[-0.28801,0.033943]	0.8	-0.002002[-0.005768,0.001762]	-0.62
**No risk**	1		1	
ANC visit				
**No visit**	1		1	
**1–3 visit**	0.06289**[0.5648,0.06930]	19.7	0.01247[-0.001681,0.02662]	3,91
**≥visit**	0.10003**[0.091828,0.10823]	31.4	0.01233[-0.0009666,0.02563]	3,86
Parity				
**1**	1		1	
**2–4**	-0.00004746[-0.0001966,0.0001017]	-0.01	-0.001623[-0.01483,0.01157]	-0.5
**≥5**	0.0001632[-0.0004925,0.0008191]	0.05	0.01374[-0.002762,0.03024]	4.3
Place of delivery				
**Home**	1	6.3	1	-0.6
**Health facility**	0.02021**[0.01239,0.02803]		-0.0025273[-0.33714,0.028654]	
Pregnancy wanted				
**Not wanted**	1	0.41	1	3.4
**Wanted**	0.001322[-0.003072,0.005716]		0.01085[-0.01753,0.03924]	
Residence				
**Rural**	1	0.6	1	-0.04
**Urban**	0.001937*[0.0002261,0,003649]		-0.000136[-0.00315,0.003430]	
Distance to health facility				
**Big problem**	1	1	1	-1,41
**Not a big problem**	0.003402*[0.0002357,0.006569]	1.1	-0.004507[-0.03533,0.2631]	
Healthcare decision making autonomy				
**Yes**	0.002796[-0.002074,0.007668]	0.87	0 .02456*[0.004033,0.04509]	7.70
**No**	1		1	

*: p-value<0.05

** p-value<0.001

#### Difference due to the effect of characteristics (coefficient)

Keeping the role of change in composition characteristics constant, 24.1% of the increase in HIV testing uptake was significantly attributed to the difference in the effects of the characteristics between 2005 and 2016. Health care decision making(7.7%) was the only factor that showed a significant contribution for the increase in the percentage of HIV testing uptake (**Table 3**).

### Spatial distribution of low proportion of HIV testing uptake among pregnant women in Ethiopia

A total of 525,592 and 612 clusters were considered for the spatial analysis of areas with low proportion HIV testing uptake among pregnant women in 2005, 2011 and 2016 EDHS respectively. Each point on the map represents one enumeration area with low proportion HIV testing uptake in each cluster. The **red color** indicates areas with higher proportion of low proportion of HIV testing uptake of 84% and more whereas green color indicates EAs with lower proportion of low proportion HIV testing uptake.

**Fig 2:**
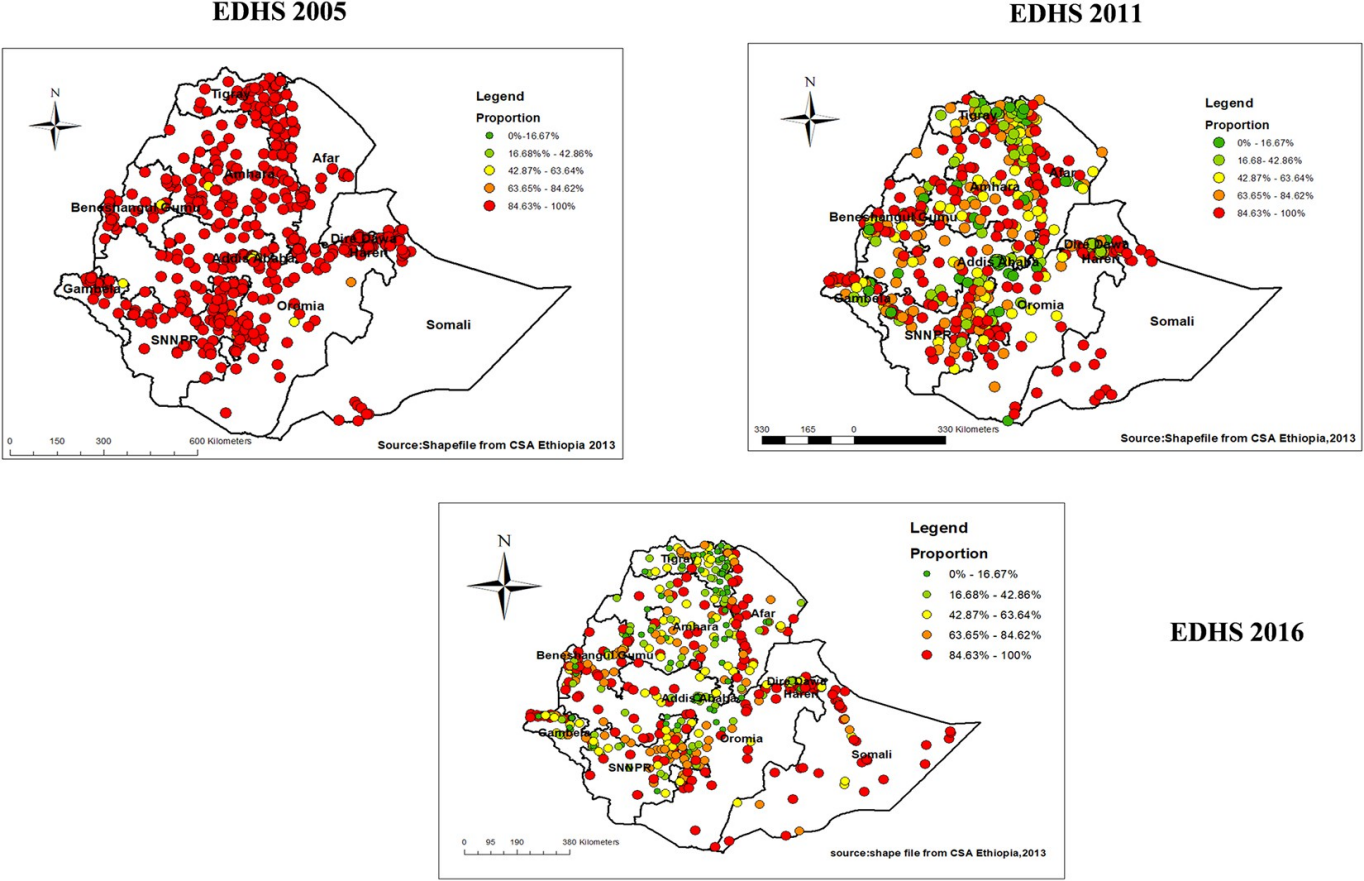
Spatial distribution of low proportion HIV testing uptake among pregnant women in Ethiopia, 2005, 2011, 2016.

Higher proportion of low proportion HIV testing uptake among pregnant women occurred in almost all parts of the country including Tigray, Amhara, Oromia, central part of Dire Dawa and Harari, North and east part of SNNPRs and some part in Southern Somali in 2005 (**Fig 2**). While in 2011 EDHS the Higher proportions of low proportion HIV testing uptake among pregnant women were aggregated mainly in some part of Afar, most parts of SNNPRs, parts of Amhara, Tigray, central Benishangul-Gumuz, Northern Gambella, Southern Somali (**Fig 2**). This low proportion of HIV testing uptake showed decrement specially in Amhara and Tigray regions in 2016 EDHS, and with higher distribution in borders of Afar with Amhara, some parts of Benishangul-Gumuz, Gambella and central part of Dire Dawa and Harari, the distribution further expanded to include most parts of Somali in 2016 (**Fig 2**).

### Spatial autocorrelation

**Fig 3:**
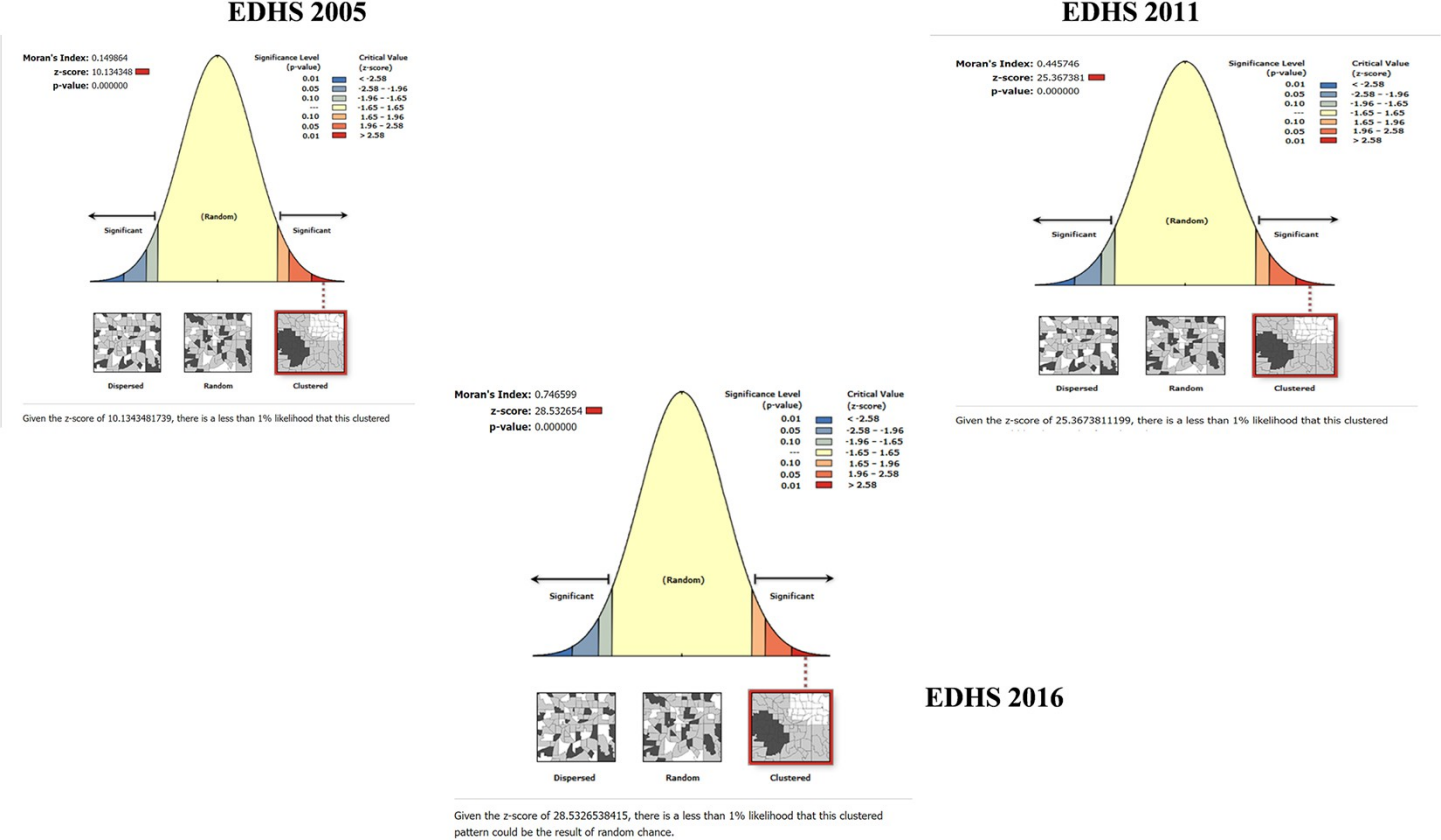
Spatial autocorrelation analysis of low proportion HIV testing uptake among pregnant women in Ethiopia, 2005, 2011, 2016 EDHS.

The spatial distribution of low proportion HIV testing uptake among pregnant women was found to be spatially clustered in all three EDHS’s, with Global Moran’s I 0.149864 (p-value = 0.00), 0.445746(p-value = 0.00),0,746599(p-value = 0.00) in 2005,2011 and 2016 EDHS respectively indicating a significant clustering (**Fig 3**).

### Hot and cold spot analysis

**Fig 4:**
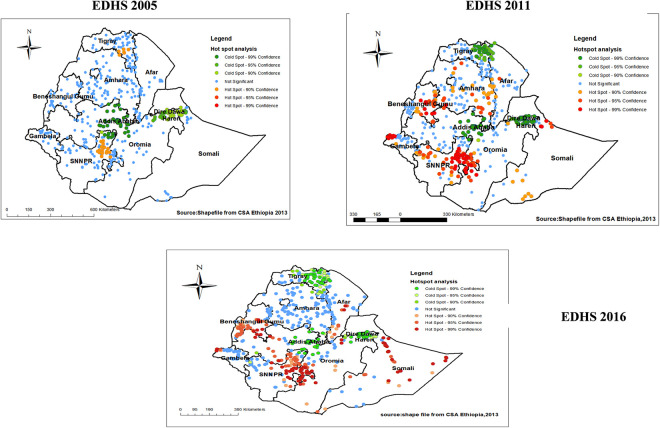
Hotspot and cold spot analysis of low proportion HIV testing uptake among pregnant women in Ethiopia, 2005, 2011, 2016 EDHS.

The Getis- Hot spot analysis identified hot spot and cold spot for each survey. In each Hot spot analysis, the **red color** indicates that significant hotspot areas (higher proportion areas of low proportion HIV testing uptake), which were found in north Tigray and north east part of SNNPRs in the 2005 EDHS, while the spatial clustering of hotspot areas(high risks) were consistent in Southern and central part of Ethiopia through 2011 and 2016. Specifically, in 2011 the hotspot areas further expanded to include central Benishangul-Gumuz, northeast part of SNNPRs, north Gambela and some part of Amhara, and south Somali. The hotspot analysis in 2016 also revealed, high proportions were mostly clustered central Benishangul-Gumuz, northeast part of SNNPRs north, central Somalia and some parts in Oromia, and Afar. On the other hand, the **green color** indicates significant cold spot areas (lower proportion of low proportion HIV testing uptake), mostly clustered in Addis Ababa, central part of Dire Dawa and Harari in 2005. In 2011 and 2016 cold spot areas were consistently clustered in Addis Ababa, central part of Tigray and Dire Dawa and Harari (**Fig 4**).

### Interpolation

**Fig 5:**
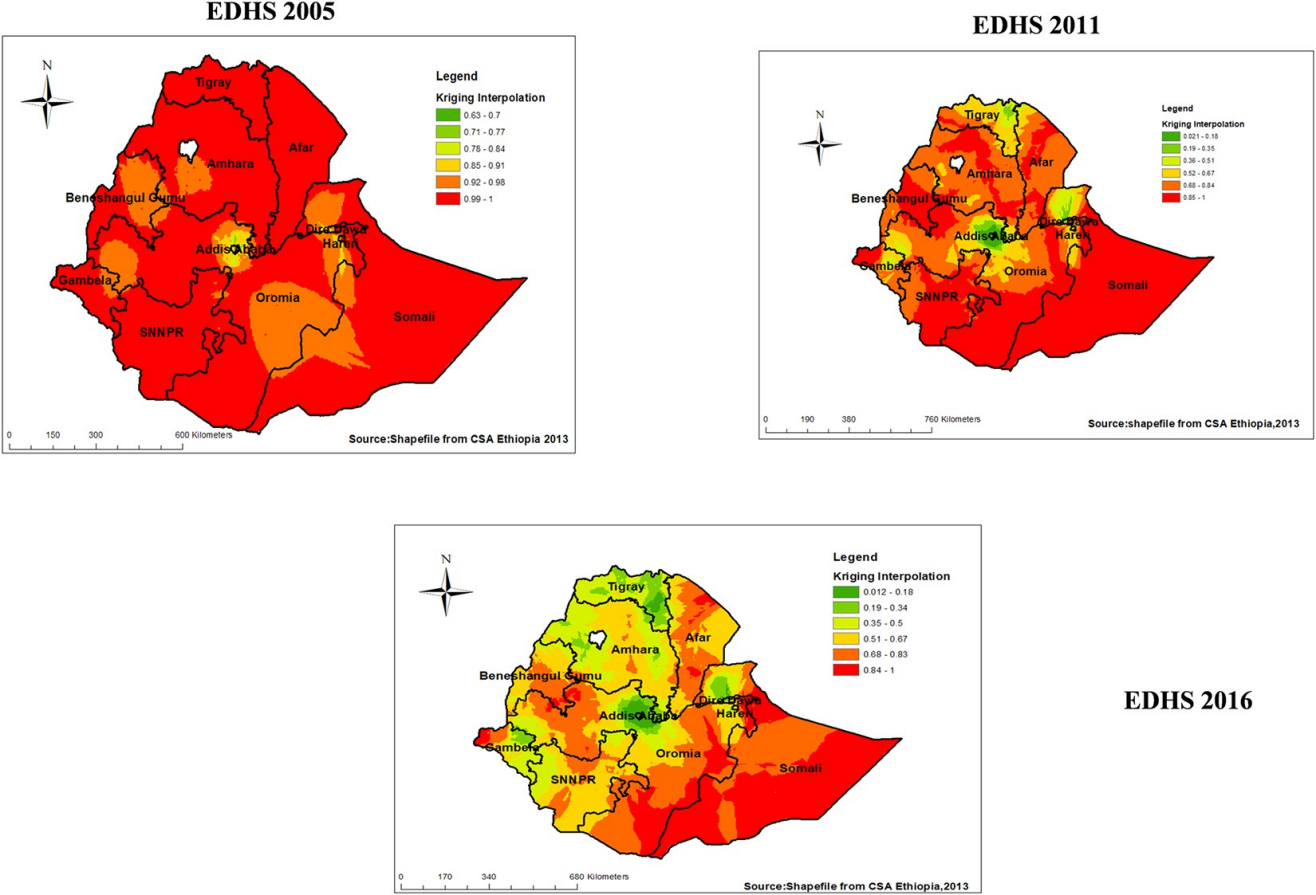
Kriging interpolation of low proportion HIV testing uptake among pregnant women across regions in Ethiopia, 2005, 2011, 2016 EDHS.

Overall across the three waves of EDHS, the prediction map revealed there was noticeable geographical variation. In 2005 EDHS almost all parts of Ethiopia were detected as predicted areas to have low proportion HIV testing uptake, except for the predicted low risk areas in Dire-Dewa and Adiss Abeba (**Fig 5**). While in 2011 EDHS except for most parts of Tigray, Adiss Abeba, Dire-Dawa and Harari Gambella with predicted low risk areas, most regions in the country had predicted high risk for low proportion HIV testing uptake among pregnant women (**Fig 5**). The interpolation in 2016 EDHS depict a predicted low risk regions of HIV testing uptake in most part of Ethiopia, whereas predicted high risk areas of HIV testing uptake in most parts of Somali, central and Southern Afar, parts of SNNPR, Benishangul-Gumuz, Western Gambella and borders of Oromia with Somali(**Fig 5**).

#### Spatial sat scan analysis (Bernoulli based model)

**Fig 6:**
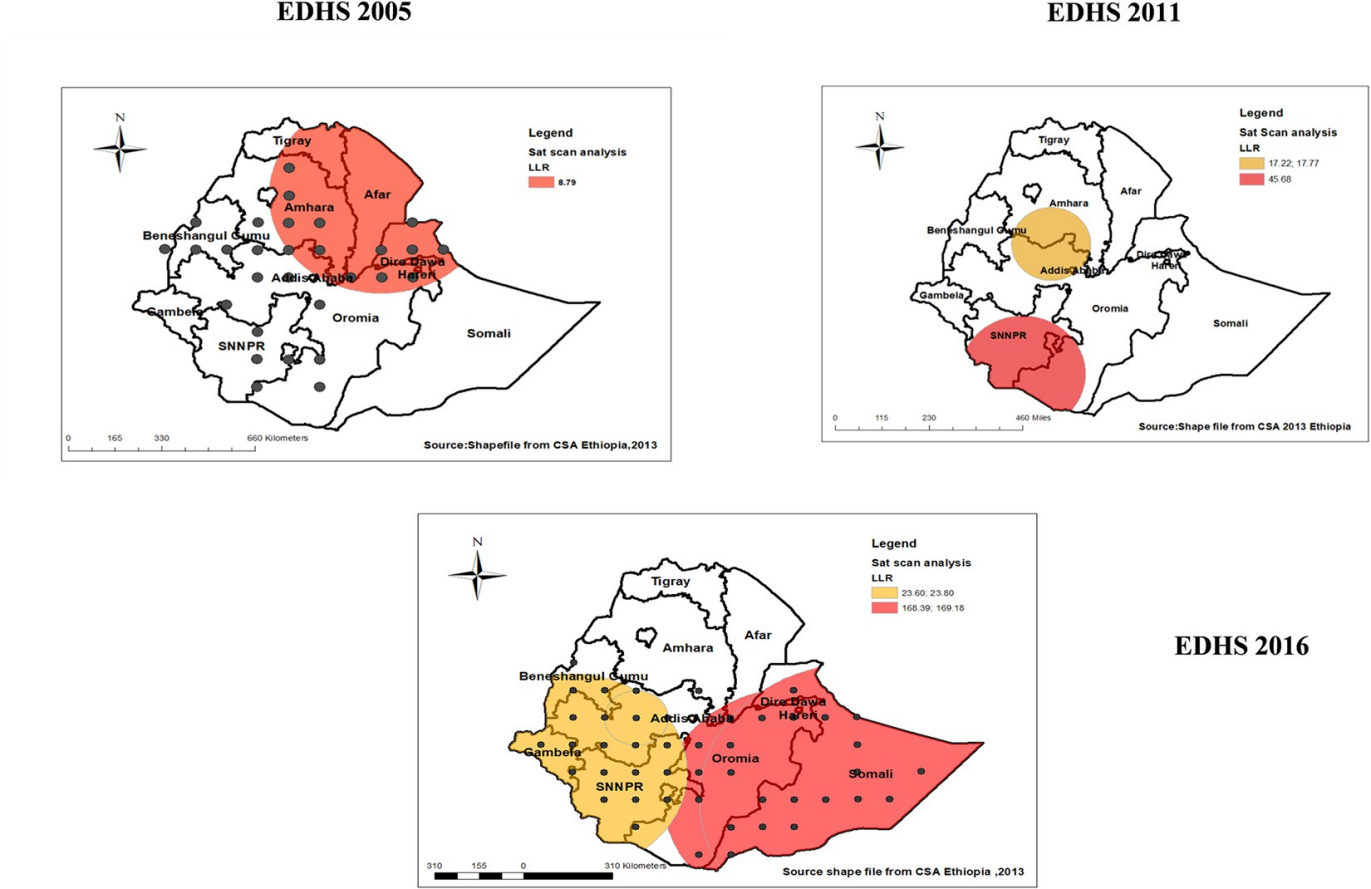
Spatial SaT scan of low proportion HIV testing uptake among pregnant women in Ethiopia, 2005, 2011, 2016 EDHS.

The result from spatial kuldorff”s scan analysis in 2005 depicted three spatial clusters but only one was statistically significant, most parts of Amhara, Afar and Dire-Dewa were identified as the most likely (primary clusters) with low proportion HIV testing uptake among pregnant women in Ethiopia represented in red color ring (**Fig 6**). The window was centered at 8.000000 N, 34.000000 E of geographic location with 398.97 km radius, and Log-Likelihood ratio (LLR) of 8.79, at p-value < 0.001, and was detected as the most likely cluster with maximum LLR. It showed that pregnant women within this spatial window had 1.01 times higher likelihood of having low proportion HIV testing uptake than women outside the window.

Among the five spatial clusters revealed in EDHS 2011, only three were statistically significant clusters. Accordingly, the red circular spatial window (**Fig 6**) which was the primary cluster encompassed SNNPRs, detected as the most likely cluster with maximum LLR of 45.68, 33.69 and 17.77. It showed that women within this spatial window had 1.19 times higher likelihood of having low proportion of HIV testing uptake than women outside the window.

While in 2016 EDHS spatial scan analysis six spatial clusters were visible, and only four were statistically significant. Most likely primary clusters and secondary clusters of low proportion HIV testing uptake in Ethiopia were also identified. The red spatial windows (**Fig 6**) covers the primary cluster which was located in most parts of Somali, borders Oromia with Somalia, it was detected as the most likely cluster with maximum LLR 169.18, 168.39. It revealed that pregnant women within this spatial window had 1.47 times higher likelihood of having low proportion HIV testing uptake than pregnant women outside the window.

### Factors affecting the spatial variation of HIV testing uptake among pregnant women (modeling spatial relationships), EDHS 2016

#### Ordinary least square analysis

The OLS model explained about 67.1% (Adjusted R square = 0.671) of the spatial variation of low proportion of HIV testing uptake among pregnant women with AICc value of -302.8 in EDHS 2016. All the assumption for the OLS was checked, but not all were met. The Joint Wald statistic and Joint F-Statistic were statistically significant (p< 0.001) and this shows that the overall model was significant and also there was no Multicolinearity between explanatory variables (Variance inflation factor (VIF) < 7.5). In addition, the Spatial Autocorrelation test for residuals revealed that residuals were not spatially auto correlated(Moran’s I = -0.016849, P-value = 0.5607). Furthermore, Jarque-Bera Statistic were statistically significant depicting that residuals were not normally distributed, finally the Koenker (BP) Statistic was also significant(p< 0.001),indicating relationships between some or all of the explanatory variables and dependent variables is non-stationary which reveals the difference of coefficients across enumeration areas. In the context of this finding Koenker (BP) test proved significant, enabling the performance of geographically weighted regression. The robust probability was used to determine the statistically significant of the coefficients, accordingly the coefficients of women with no ANC visit, women with no MTCT knowledge, women with no media exposure, and women with previous health facility delivery were statistically significant (p< 0.01) and associated with low proportion of HIV testing uptake among pregnant women in EDHS 2016 (**S2 Table**).

#### Geographically weighted regression

The OLS regression identified predictors of low proportion HIV testing uptake hotspots. The GWR improves the model fit when the relationship between the predictors and HIV testing uptake among pregnant women is stationary across regions. Thus, it depicts significant improvement over OLS.

The Adjusted R-Squared increased from 0.671 (67.1%) to 0.694(69.4%), suggesting that GWR better explained the geographical disparity of HIV testing uptake. The AICc value also decreased from -302.8 in the OLS model to -317.5 in the GWR model (**S2 Table**). Thus, higher adjusted R square and lower Akaike’s Information Criterion (AICc) value obtained from the GWR model (as compared to the OLS model) helps us to move to local regression model GWR. In the geographically weighted regression analysis, all the explanatory variables included in the OLS model were considered. The strength of the relationship with independent variables varied spatially and variable effects had both positive and negative impact.

**Fig 7:**
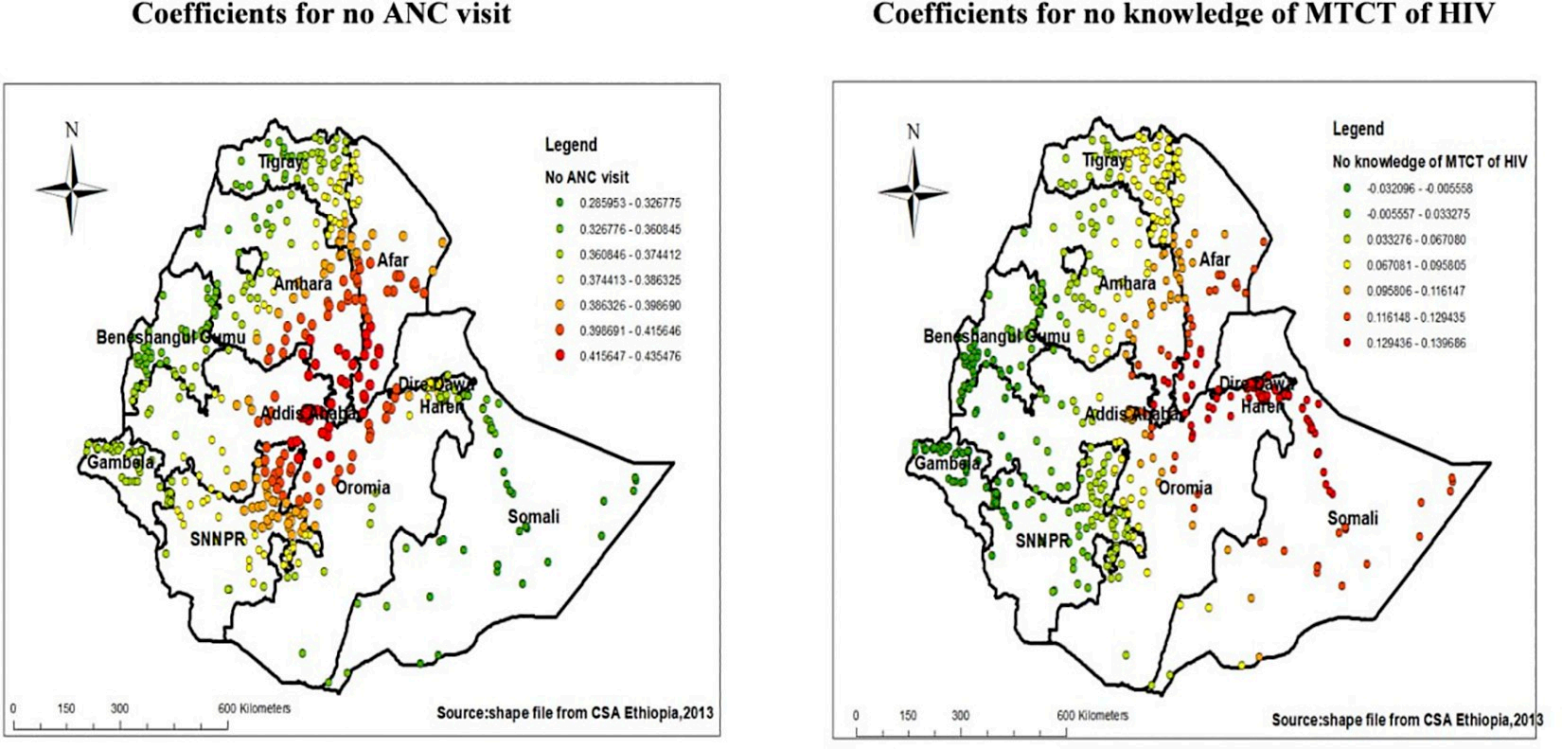
Coefficients for women who have no ANC visit and MTCT of HIV knowledge among pregnant women Ethiopia, EDHS 2016.

The result of geographically weighted regression identified different significant variable coefficients for the identified variables on ordinary least square analysis. Higher coefficients for women with no ANC visit were detected in parts of Amhara region, Afar, Oromia region, and Northern part of SNNPR *(***Fig 7**). Similarly, as shown on the right side of the figure the red-colored clustered points (found in some parts of Afar region, central Dire-Dewa and Harari, north part of Oromia, and parts of Somali indicate areas where the coefficients were largest for proportion of women with no MTCT of HIV knowledge, which in turn indicates the strong positive relationship between poor MTCT knowledge and not being tested for HIV (**Fig 7**).

**Fig 8:**
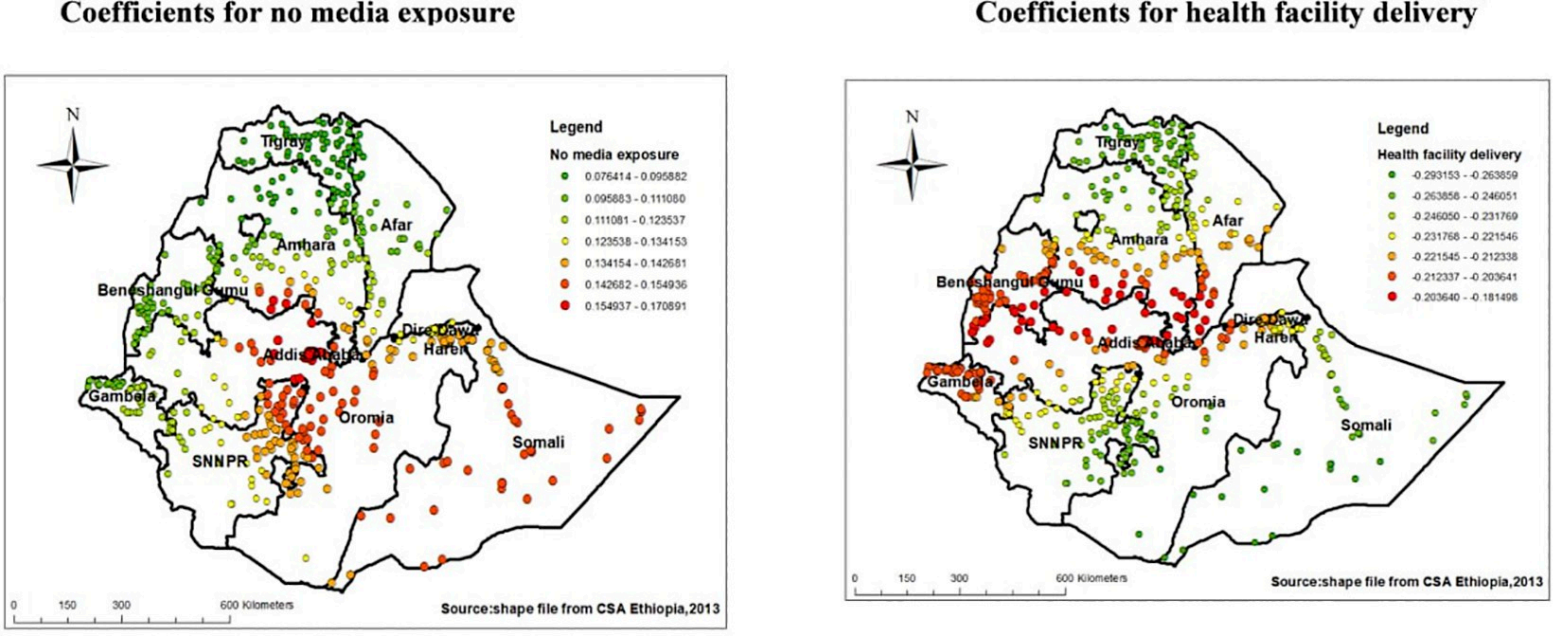
Coefficients for women who have no media exposure and health facility delivery among pregnant women Ethiopia, EDHS 2016.

As shown on the left side of the figure having no media exposure was also another factor positively associated with low proportion of HIV testing among pregnant women, the red colored clustered points shows parts of Somali, Adiss Abeba, Oromia and north Eastern SNNPRs regions had higher coefficients for low proportions of HIV testing (**Fig 8**). Another significant factor having negative relationship with low proportion of HIV testing uptake was health facility delivery, areas in Southern Amhara and parts of Gambella and Benshangul-Gumuz had higher coefficients, which in turn indicated women with previous health institution delivery are more likely to take HIV test (**Fig 8**).

## Discussion

This study aimed to determine the trends, factors for the changes, and spatial distribution of HIV testing uptake among pregnant women in during the time 2005–2016.

The finding of this study demonstrated the trend in HIV testing uptake has significantly increased over the study period(2005–2016) from 0.51%[0.32%, 0.77%] in 2005 to 32.4% [31.0%, 33.8%] in 2016. This finding align with similar studies in Nigeria [38], and Zambia [39] and the United Nations report [40] highlighting success of various intervention implemented in these regions [41]. The observed increment in Ethiopia overtime could be attributed to the tremendous nationally implemented HIV programs and initiatives in Ethiopia since 2007 including Behavior change initiatives, community based and health extension worker program to provide basic healthcare program like HIV testing, and the governments’ commitment to achieve Millennium Development Goals (MDG) and Sustainable Development Goals (SDGs) goals in attempt to eliminate MTCT [4, 42]. It can also be due to the improvement in healthcare infrastructures and integration of HIV testing with other maternal and child health services like antenatal care visit [43]. Achieving the global 95-95-95 targets set by UNAIDS could profoundly impact the prevention of mother-to-child transmission (MTCT) of HIV, however, this increment still falls short of the global targets [2]. Possibly this might be because healthcare accessibility is still limited in the rural areas, additionally despite the integration of HIV testing services with ANC visits in Ethiopia there are still number of pregnant women not accessing the optimal number of visits [44].

The multivariable decomposition analysis identified the significant factors that contributed to the increase in HIV testing among pregnant women the last 11 years. About 75.9% of the change in HIV testing uptake over the entire study period (2005–2016) was attributed to the change in women’s composition. The change in composition of women’s knowledge of MTCT of HIV showed a 3.3% significant positive effect on the change in experiencing HIV testing uptake among pregnant women. This finding is supported by studies conducted in East Africa [16] and East Gojjam, Ethiopia [14]. The explanation could be that having MTCT knowledge of HIV will give women trustworthy information about the risks, safety measures to take, and HIV therapies that are accessible. This will motivate women to seek HIV services early and protect the health of both mother and child. Women who comprehend the MTCT of HIV will also avoid HIV myths and testing-related anxiety, enabling them to make better decisions on whether or not to get tested for the illness [45].

Moreover, an increase in composition of pregnant women who have HIV counseling revealed a 10.2% significant positive effect on the increment of HIV testing uptake among pregnant women over time. The evidence from this study is consistent with a study done in Kenya [20] and Zimbabwe [19]. These might be due to the fact that HIV counseling provides pregnant women with adequate and comprehensive information about HIV, transmission and prevention methods and also provide safe and supportive environment which can provide reassurance by addressing their concern [46].

Rise in the proportion of pregnant women who has one or more ANC visit revealed a 19.7% indispensable positive effect on the increment of HIV testing uptake among pregnant women. Similarly pregnant women with four or more ANC visit had a 31.4% significant positive effect on the increment of HIV testing uptake among. This finding is supported by studies done in Malawi [47] and a systematic review done in Ethiopia [48]. This is because of HIV testing has been integrated as part of ANC obstetric care at different levels of the health care system as an opt-out strategy provides a window opportunities for early detection of HIV and PMTCT [4]. Thus, the more often a pregnant woman comes in contact with antenatal care, the more likely a women will be exposed to information on MTCT/PMTCT which influence their decision to uptake testing [49].

An increase in composition of women who deliver at a health institution revealed a 6.3% significant positive effect on the increment of HIV testing uptake among pregnant women. This evidence is consistent with a finding [48]. The possible explanation for these could be pregnant women who have previously given birth in a health care facility may develop a trusting relationship with healthcare professionals, which in turn encourages them to request HIV testing services during subsequent pregnancies. In addition, women who had previously given birth in a healthcare facility may have received health counseling and education during earlier ANC visits [50].

Although the compositional contribution was small, 0.6% of the increment in HIV testing uptake among pregnant was accounted by the increase in the proportion of women residing in urban areas. This finding is consistent with the studies done in Nigeria [38] and Uganda [51]. This might be due to the fact that health facilities, in particular, are more readily accessible in urban areas than in rural ones, giving pregnant women there more opportunities to seek testing. Aside from this, urbanization is also associated with a better rate of literacy and access to educational resources ad media outlets, which improves awareness of HIV, how it spreads, and the value of testing [52].

Furthermore, not perceiving distance to health facility as a problem was another significant factor associated with the increment of HIV testing uptake among pregnant women. This result is supported by a study done in east Africa [53]. The reason for this might be as a result of many initiatives health care facilities are being more accessible, this increment may result in shorter travel time. Additionally the increment in urbanization minimizes the barriers in transportation making it less difficult to reach health facilities for HIV testing [54].

One fourth of the increase in HIV testing uptake during the overall study period (2005–2016) was due to change in coefficient, keeping the effect of compositional factors constant. It is consistent with a study done in Nigeria [55]. This might be explained by, through years women’s empowerment and decision making autonomy have been recognized to be vital to women’s access to reproductive healthcare, including HIV services and other reproductive and sexual health issues thus, It improves women’s self-assurance to test for HIV, identify their HIV status, and be able to avert mother-to-child transmission [56].

The findings of this study also showed significant spatial variation across regions in Ethiopia in the three waves of EDHS. The Hot spot areas with low proportion HIV testing uptake in 2005 were found in north Tigray and north east SNNPRs then shifted to central Benishangul-Gumuz, north east SNNPRs, north Gambella, central Somali and some parts of Amhara, while the hotspot areas stayed consistent in 2016 with Expansion of hotspot areas in Somali and parts of Oromia. These areas are consistent with the predicted maps from the spatial interpolation analysis and the SaT Scan analysis. Different studies also reported the presence of geographical variations regarding pregnant women’s HIV test uptake [57, 58]. This fluctuating trend across regions might be attributed to the inconsistent intervention and program implementation; hence effectiveness depends on region’s strength to use the program in the context of the region. Additionally, the spatial distribution of health facilities in Ethiopia shows that Benishangul-Gumuz, SNNPRs, Somali, and Gambella have shortage of facilities compared to the population size of the national average, and travels long distance to access services [59]. The variation could also be attributed to the disparity in distribution of maternal health service, disparity in cultural attitudes toward HIV testing among the regions of Ethiopia, specially People in Somali regions are predominantly pastoralists and they move from place to place to place as a result it could be difficult to provide health services [60].

In the Geographically weighted regression analysis, Lack of knowledge of MTCT of HIV, lack of ANC visit, previous delivery in a health facility and lack of media exposure were significant predictors of hotspot areas without HIV testing uptake among pregnant women in EDHS, 2016. There was a positive relationship between pregnant women who had no ANC visit and hot spots areas of low proportions of HIV testing uptake among pregnant women in parts of Amhara region, Afar, Oromia region, and Northern part of SNNPR. This might be due to missing information about HIV testing as a result of missed ANC visit as a result of barrier to the health facility accessibility due to distance or limited infrastructures in these regions and According to the report of the 2016 EDHS, women in these regions had low ANC utilization [23].

Similarly, pregnant women with no media exposure was another factor positively associated with low proportions of HIV testing uptake in Somali, Oromia and North Eastern SNNPRs regions for low HIV testing. Possible explanation for this might be these areas consists more of rural residents contributing to poor access to media which in turn leads to lack of awareness about risks and prevention mechanism of HIV transmission among pregnant women, access and choices of HIV testing mechanism for pregnant women leading to lower uptake [61].

Furthermore, parts of Afar region, central Dire-Dewa and Harari, north part of Oromia, and parts of Somali were areas having the strong positive relationship between lack of MTCT knowledge and low proportion HIV testing uptake were found. This may be as a result of differences in the availability of educational resources and information on MTCT, as these border regions struggle with infrastructural and health care accessibility issues. Pregnant women may therefore be less informed of MTCT and the advantages of HIV testing in places with poor access to health education, which in turn contributes to reduced HIV testing [61].

Another significant factor having negative relationship with low proportion of HIV testing uptake was health facility delivery, areas in Southern Amhara and afar, and parts of Gambella and Benshangul-Gumuz had higher coefficients. According to the report of the 2016 EDHS, women in this regions are associated with low number of health institution delivery [23], which in turn minimizes the exposure they have with health professionals providing them with lower health education about benefit of HIV testing during pregnancy contributing to the lower numbers of HIV testing uptake among pregnant [62].

## Strength and limitations of the study

Though the current study has its own strengths, like it was based on a large datasets representing the whole country Ethiopia and incorporated data from three successive surveys, there were some limitations like: Variables like access and availability of HIV testing were not addressed in this study because these variables were not available in EDHS and Some variables were not consistently collected in all EDHS surveys like stigma indicators, thus these variables were not used for the decomposition analysis. Moreover, there was a possibility of committing social desirability and recall bias in ascertaining HIV testing, although it was claimed that strong efforts were made to minimize it mainly through extensive training of data collectors, recruiting experienced data collectors and supervisors.

In addition The SaT Scan analysis detects only circular clusters; irregularly shaped clusters may not be identified, this might be the reason inclusion of Dire-Dewa which was a cold spot surrounded by hotspot as a significant cluster in 2016 EDHS and Afar in 2005 which was insignificant spot. The displacement of geographical coordinates for confidentiality might affect estimates of spatial result. Therefore, the interpretation or conclusion based on this study should consider these limitations.

## Conclusions

In this study, trends of proportion of HIV testing uptake among pregnant women revealed significant increment overtime (2005–2016) in Ethiopia but with 2.9% annual rate of increment the country might be far from achieving global targets The principal contributing factors for the change were knowledge of MTCT of HIV, HIV counseling, Number of ANC visit, previous place of delivery, residence, distance to health facility and health care decision making autonomy. The spatial distribution of low proportion of HIV testing among pregnant women identified consistent hot-pot areas in Tigray and SNNPRs in 2005 which later shifted to most parts of Benishangul-Gumuz, Somali, SNNPR, Gambella and Oromia were in 2016. Lack of knowledge of MTCT of HIV, lack of ANC visit, health facility delivery and absence of media exposure were significant predictors of hotspot areas with low proportion HIV testing uptake among pregnant women in EDHS, 2016. Therefore, the Ethiopian Ministry of Health and the regional health bureaus of Ethiopia better give emphasis to high risk areas like Benishangul-Gumuz, SNNPRs Somali, Oromia, and Gambella to design local based intervention strategies tailored to unique social and economic context of regions to mobilize women to use maternal health service like HIV testing and ANC visits.

## Supporting information

S1 Table Trends in HIV testing uptake rate among women’s who gave birth in the last two years prior to the survey by selected characteristics 2005, 2011, and 2016 Ethiopia Demographic and Health Surveys. (DOCX)

S2 Table Summary of OLS results and diagnostics for low proportion of HIV testing uptake among pregnant women in Ethiopia, EDHS 2016. (DOCX)

## List of abbreviations/acronym

AIDS: Acquired Immunodeficiency Syndrome
ANC: Antenatal Care
ART: Anti-retroviral Treatment
CDC: Centers for Disease Control and Prevention
CI: Confidence Interval
DHS: Demographic Health Survey
EDHS: Ethiopian Demographic Health Survey
HIV: Human Immune-Deficiency Virus
GWR: Geographically Weighted Regression
MTCT: Mother To Child Transmission
OLS: Ordinary Least Square
PMTCT: Prevention of Mother to Child Transmission
UNAIDS: Joint United Nations Programme on HIV/AIDS
SNNPRs: Southern Nations and Nationality People Regional state
WHO: World Health Organization
