# Supplementary material for: Trend, spatial distribution, and factors associated with HIV testing uptake among pregnant women in Ethiopia, based on 2005–2016 Ethiopia demographic and health survey: A multivariate decomposition analysis and geographically weighted regression
Source: PLoS One. 2024 Oct 4;19(10):e0308167. doi: 10.1371/journal.pone.0308167 (PMC11451988; doi:10.1371/journal.pone.0308167)
Supplement: S1 Table — (DOCX) [file pone.0308167.s001.docx]

Trends in HIV testing uptake rate among women’s who gave birth in the last two years prior to the survey by selected characteristics 2005, 2011, and 2016 Ethiopia Demographic and Health Surveys.

| Characteristics | 2005 N=4321 | 2011 N=4453 | 2016 N=4246 | point difference in HIV testing uptake | | |
| --- | --- | --- | --- | --- | --- | --- |
|  |  |  |  | Phase 1 2011-2005 | Phase 2 2016-2011 | Phase 3 2016-2005 |
| Age | | | | | | |
| 15-19  20-24  25-29  30-34  ≥35 | 0.2  0.7  0.7  0.3  0.5 | 18.2  26.3  20.3  17.6  15.1 | 30.5  34.8  34.0  32.5  27.8 | 18.0  25.6  19.6  17.3  14.6 | 12.3  8.5  13.7  14.9  12.7 | 30.4  34.1  33.6  32.2  27.3 |
| Marital status | | | | | | |
| Single  Married  others | 0.4  0.4  2.7 | 28.9  19.6  24.6 | 47.0  32.5  27.5 | 28.5  19.2  21.9 | 18.1  12.9  2.9 | 46.6  32.1  24.8 |
| Women’s education status | | | | | | |
| No education  Primary  Secondary and above | 0.2  0.9  5.0 | 11.7  29.1  83.2 | 21.7  41.1  75.0 | 11.5  28.3  78.2 | 10.0  12.0  -8.2 | 21.5  40.2  70.0 |
| Paternal education status | | | | | | |
| No education  Primary  Secondary and above | 0.2  0.3  2.5 | 12.0  21.1  58.4 | 23.9  31.7  64.2 | 11.8  20.8  55.9 | 11.9  10.6  5.8 | 23.7  31.4  61.7 |
| Occupation status | | | | | | |
| Working  Not working | 0.8  0.4 | 21.3  18.7 | 37.5  28.8 | 20.5  18.3 | 16.2  10.1 | 36.7  28.4 |
| Religion | | | | | | |
| Orthodox  Muslim  others | 0.7  0.5  0.2 | 26.9  18.1  12.8 | 50.3  21.2  26.3 | 26.2  17.6  12.6 | 23.4  3.1  13.5 | 49.6  20.7  26.1 |
| Residence | | | | | | |
| Rural  Urban | 0.2  4.1 | 13.5  60.9 | 26.3  76.6 | 13.3  56.8 | 12.8  15.7 | 26.1  72.5 |
| Wealth index |  |  |  |  |  |  |
| Poorest  poorer  Middle  Richer  Richest | 0.0  0.3  0.4  0.2  2.0 | 6.7  10.3  12.8  21.8  60.1 | 14.3  23.2  27.2  40.1  70.5 | 6.7  10.0  12.4  21.6  58.1 | 7.6  12.9  14.4  18.3  10.4 | 14.3  22.9  26.8  39.9  68.5 |
| Media exposure | | | | | | |
| Yes  No | 1.1  0.2 | 27.7  8.9 | 49.0  23.6 | 26.6  8.7 | 21.3  14.7 | 47.9  23.4 |
| Knowledge of MTCT of HIV | | | | | | |
| Yes  No | 1.5  0.2 | 24.8  14.8 | 41.8  21.4 | 23.3  14.6 | 17.0  6.6 | 40.3  21.2 |
| Comprehensive knowledge of HIV | | | | | | |
| Low  High | 0.5  0.5 | 20.2  19.1 | 33.5  29.6 | 19.7  18.6 | 13.3  10.5 | 33.0  29.1 |
| Risky sexual behavior | | | | | | |
| No risk  With risk | 0.3  1.8 | 19.4  22.1 | 30.8  39.1 | 19.1  20.3 | 11.4  17.0 | 30.5  37.3 |
| HIV Counseling | | | | | | |
| Yes  No | 18.8  0.2 | 83.3  10.0 | 81.8  17.3 | 64.5  9.8 | -1.5  7.3 | 63.0  17.1 |
| Early sexual initiation | | | | | | |
| <18 years  ≥18 years | 0.4  0.6 | 29.1  18.7 | 30.0  36.7 | 28.7  18.1 | 0.9  18.0 | 29.6  36.1 |
| Distance to Health facility | | | | | | |
| Big problem  Not a big problem | 0.3  1.2 | 13.8  38.0 | 22.7  47.2 | 13.5  36.8 | 8.9  9.2 | 22.4  46.0 |
| Parity | | | | | | |
| 1  2-4  ≥5 | 1.1  0.6  0.2 | 32.7  21.0  12.6 | 44.6  36.2  21.4 | 31.6  20.4  12.4 | 11.9  15.2  8.8 | 43.5  35.6  21.2 |
| Place of delivery | | | | | | |
| Home  Health facility | 0.2  4.9 | 13.8  65.2 | 15.8  59.4 | 13.6  60.3 | 2.0  -5.8 | 15.6  54.5 |
| Pregnancy wanted | | | | | | |
| Wanted  Not wanted | 0.5  0.4 | 20.3  17.7 | 33.6  18.9 | 19.8  17.3 | 13.3  1.2 | 33.1  18.5 |
| Number of ANC | | | | | | |
| No visit  1-3 visit  ≥4 visit | 0.03  1.1  2.6 | 0.1  37.7  58.4 | 0.1  37.6  61.8 | 0.1  36.6  55.8 | 0.0  -0.1  3.4 | 0.1  36.5  59.2 |

*ANC: antenatal care; others*: protestant catholic/traditional; other*: Widowed, separated, divorced
