# Supplementary material for: Trend, spatial distribution, and factors associated with HIV testing uptake among pregnant women in Ethiopia, based on 2005–2016 Ethiopia demographic and health survey: A multivariate decomposition analysis and geographically weighted regression
Source: PLoS One. 2024 Oct 4;19(10):e0308167. doi: 10.1371/journal.pone.0308167 (PMC11451988; doi:10.1371/journal.pone.0308167)
Supplement: S2 Table — (DOCX) [file pone.0308167.s002.docx]

Summary of OLS results diagnostics and GWR for low proportion of HIV testing uptake among pregnant women in Ethiopia, EDHS 2016

| Variable | Coefficients | Standard error | t-statistics | Probability | Robust standard error | Robust statistic | Robust probability | | VIF |
| --- | --- | --- | --- | --- | --- | --- | --- | --- | --- |
| Intercept | 0.2146 | 0.058600 | 3.6625 | 0.000284* | 0.062098 | 0.456244 | 0.000601* | | ---- |
| Proportions of women with no ANC | 0.339103 | 0.039509 | 8.5828 | 0.000000* | 0.039153 | 8.660936 | 0.000000* | | 2.55 |
| Proportions of women with low HIV knowledge | 0.019403 | 0.034581 | 0.5610 | 0.574967 | 0.035257 | 0.550322 | 0.582312 | | 1.08 |
| Proportions of women no HIV counseling | 0.274947 | 0.033018 | 8.3271 | 0.594667 | 0.039640 | 6.936146 | 0.467899 | | 2.09 |
| Proportions of women with no Education | 0.041917 | 0.032308 | 1.2974 | 0.194985 | 0.035728 | 1.173247 | 0.241163 | | 2.24 |
| Proportions of women with no Media Exposure | 0.117504 | 0.034616 | 3.3945 | 0.000748* | 0.045678 | 2.572429 | 0.010331* | | 2.99 |
| Proportions of women with no knowledge of MTCT of HIV | 0.061762 | 0.029528 | 2.0916 | 0.036881* | 0.034386 | 1.796166 | 0.002977* | | 1.33 |
| Proportions of women with Health facility delivery | -0.262180 | 0.038107 | -6.8801 | 0.000000* | 0.044329 | -5.914469 | 0.000000* | | 3.84 |
| OLS diagnostics | | | | | | | | | |
| Number of observation | | 612 | | Akaike’s Information Criterion (AICc) | | | | -302.8 | |
| Multiple R-Squared | | 0.693 | | Adjusted R-Squared | | | | 0.671 | |
| Joint F-Statistic | | 52.289544 | | Prob(>F), (13,598) degrees of freedom | | | | 0.000** | |
| Joint Wald Statistic | | 3408.702295 | | Prob(>chi-squared),(13) degrees of freedom | | | | 0.000** | |
| Koenker (BP) Statistic | | 60.819939 | | Prob(>chi-squared),(13) degrees of freedom: | | | | 0.000** | |
| Jarque-Bera Statistic | | 117.576142 | | Prob(>chi-squared),(2) degrees of freedom | | | | 0.000** | |

*VIF: Variance inflation factor; ******p-value<0.05; ******p-value<0.001; ANC: Antenatal care visit

Summary of geographically weighted regression (GWR) model with low proportion of HIV testing uptake among pregnant women in Ethiopia, EDHS 2016

| Residual squares | 18.9 |
| --- | --- |
| Effective number | 43.0 |
| Sigma | 0.182 |
| Akaike’s Information Criterion (AICc) | -317.5 |
| Multiple R-Squared | 0.704 |
| Adjusted R-Squared | 0.694 |
